# ConcreteCARB: A comprehensive image dataset of concrete carbonation for computer vision tasks

**DOI:** 10.1016/j.dib.2026.112493

**Published:** 2026-01-20

**Authors:** José A. Guzmán-Torres, Sandra del C. Arguello-Hernández, Francisco J. Domínguez-Mota, Gerardo Tinoco-Guerrero, Elia M. Alonso-Guzmán

**Affiliations:** Civil Engineering Faculty, Universidad Michoacana de San Nicolás de Hidalgo, Morelia, Michoacán, 58030, Mexico

**Keywords:** Carbonation detection, Phenolphthalein test, Carbonation analysis, Artificial intelligence, Durability, Benchmark dataset

## Abstract

The ConcreteCARB dataset provides a comprehensive repository of 903 high-resolution images of concrete surfaces evaluated using the phenolphthalein test for carbonation detection. This data was collected under controlled laboratory conditions and aims to support artificial intelligence applications in civil engineering, especially in structural health monitoring tasks. The images are systematically organized into two distinct classes: “Carbonated Samples” and “No Carbonation Presence,” enabling binary classification approaches. All samples were manually tested, split, and visually labelled by expert engineers to ensure reliable ground-truth classification, in accordance with standardized procedures. The dataset includes images of concrete prism elements fabricated with varying mix designs, incorporating different water-cement ratios and additives, such as industrial silica waste and natural admixtures derived from Opuntia ficus-indica. The specimens were subjected to natural atmospheric carbonation conditions for 180 days, and their carbonation fronts were revealed by phenolphthalein staining. The samples were then split manually with a chisel and hammer, and photographic documentation was performed with a Samsung SM-S901U1 smartphone using predefined settings to ensure consistency and quality across the dataset. ConcreteCARB is intended for researchers, engineers, and data scientists working on machine learning, deep learning, and computer vision solutions for concrete diagnostics. It provides valuable training and benchmarking data for the development of automated detection, classification, and segmentation models for carbonation damage assessment. Furthermore, the dataset can serve as a foundational tool for cross-comparative studies on the efficacy of AI techniques in materials degradation analysis. The openly accessible nature of the dataset through a public repository supports reproducibility and encourages the extension of AI applications in concrete durability and sustainability studies.

Specifications Table

**Table:** 

Subject	Computer Sciences, Engineering & Materials science
Specific subject area	Artificial Intelligence, Computer Vision and Pattern Recognition, Structural Health Monitoring, Concrete degradation.
Type of data	Analyzed, Filtered, Processed. Images (.jpg)
Data collection	The authors collected data at the “Ing. Luis Silva Ruelas” materials lab at the Universidad Michoacana de San Nicolás de Hidalgo, using a Samsung SM-S901U1 smartphone for image capture and manual chisel-hammer tools for sample fracturing. Carbonation was assessed using the phenolphthalein test, as per NMX-C-515-ONNCCE-2016. Domain experts applied visual labels. All images were saved in JPG format under ambient light, without flash, at a resolution of 3000 × 4000.
Data source location	• Institution: Materials laboratory “Ing. Luis Silva Ruelas” located at the Civil Engineering Faculty, Universidad Michoacana de San Nicolás de Hidalgo. • City: Morelia, Michoacán. • Country: México. • Latitude and longitude for collected samples: 19°41′22.6″N 101°12′14.8″W.
Data accessibility	Repository name: Mendeley Data identification number: 10.17632/yxsx2v95dw Direct URL to data: https://data.mendeley.com/datasets/yxsx2v95dw/2
Related research article	None

## 1 Value of the Data

• This dataset contains 903 high-resolution images of laboratory-prepared concrete samples, manually classified into two categories: Carbonated Samples and No Carbonation Presence. It provides a valuable resource for training and validating neural networks in classification, detection, and segmentation tasks related to structural health monitoring.
• Researchers, engineers, and scientists can leverage this dataset in machine learning, deep learning, and computer vision applications to assess the condition of concrete surfaces affected by the carbonation degradation process.
• The presence or absence of carbonation damage was determined using the phenolphthalein test, ensuring accurate ground-truth labeling. This offers an industry-relevant benchmark, bridging the gap in automated carbonation detection and supporting the development of AI-driven inspection tools.
• The dataset supports benchmarking of algorithms in terms of accuracy, precision, recall, and mean average precision at 0.5 IoU (mAP@0.5), facilitating performance comparisons across different models. The well-organized structure allows for straightforward data augmentation to generate additional training samples for specific experimental objectives.
• This dataset is optimized for binary classification (Carbonated Samples vs. No Carbonation Presence) using machine learning algorithms. Furthermore, high-definition images and clear colorimetric distinction enable the training of models to automatically detect and segment carbonation in concrete structures.
• This dataset is optimized for binary classification (Carbonated Samples vs. No Carbonation Presence) using machine learning algorithms. Furthermore, high-definition images and clear colorimetric distinction enable the training of models to automatically detect and segment carbonation in concrete structures.

## 2 Background

It is undeniable that Artificial Intelligence (AI) is increasingly transforming the fields of construction, architecture, and infrastructure maintenance. The integration of AI methods—particularly computer vision—requires access to high-quality, high-resolution image datasets that capture material degradation patterns under controlled and repeatable conditions [1,2]. Recent benchmark datasets published in Data in Brief practice have established rigorous standards for computer vision resources, emphasizing the need for structured metadata, standardized annotation formats, and clear experimental protocols to ensure reproducibility. ConcreteCARB aligns with these emerging standards by providing a fully accessible, high-resolution image set accompanied by a detailed metadata inventory and a transparent specimen-based classification protocol. This structure facilitates not only the training of robust deep learning models but also the objective benchmarking of new algorithms for automated pathology detection in civil infrastructure.

Among the most relevant deterioration processes in concrete is carbonation, a phenomenon that can be evaluated rapidly through standardized laboratory or field tests using phenolphthalein as an indicator [3]. However, visual identification and classification of carbonation damage remain challenging, especially in areas with limited accessibility or inconsistent illumination. Also, manual classification of this type of pathology can take considerable time.

The motivation for developing the ConcreteCARB dataset is to provide a clean, consistent, and shareable source of labeled imagery to support AI-based assessment of concrete durability. The theoretical foundation draws from materials science and structural engineering, while the methodological basis relies on the manual visual documentation of concrete samples subjected to carbonation testing. Each image corresponds to a specimen with controlled variations in mix composition, water-cement ratio, additive use, and specimen age, following standard testing protocols to ensure reproducibility and scientific reliability.

## 3 Data Description

### 3.1 Image dataset description

**Fig. 1:**
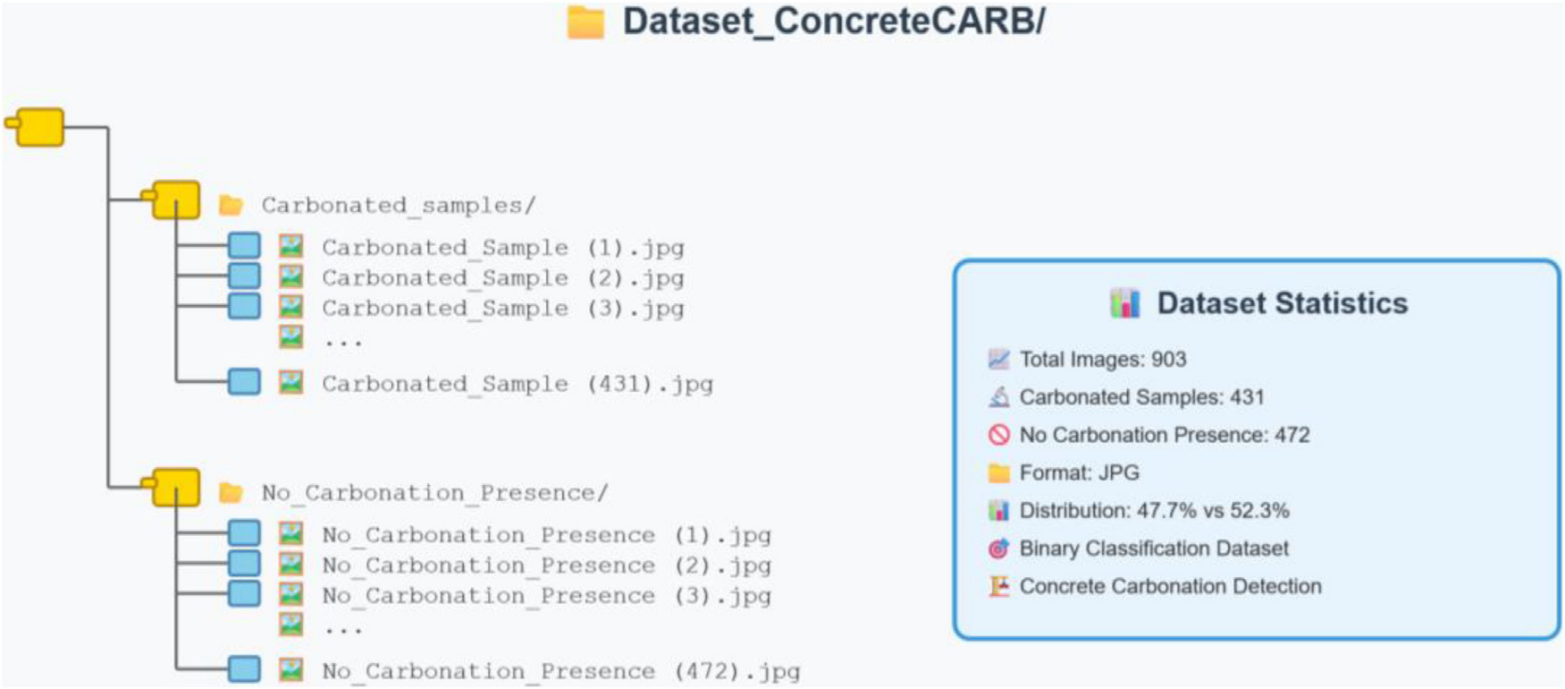
ConcreteCARB dataset structure and data statistics.

This article introduces the ConcreteCARB repository, which comprises 903 high-resolution images, organized within a main directory titled Dataset_ConcreteCARB. The repository is structured into two subdirectories: “Carbonated Samples” and “No_Carbonation_Presence”, as illustrated in Fig. 1.

ConcreteCARB repository comprises two classes: Carbonated Samples, with 431 images (47.7 %), and No Carbonation Presence, with 472 images (52.3 %). All images are provided in JPG format, ensuring accessibility and compatibility across platforms. File dimensions are variable, ranging from 669 × 669 to 4000 × 3000 pixels, with the latter being the predominant. A detailed inventory of the dataset structure, file counts, and formats is summarized in Table 1.

**Table 1:** Dataset inventory and file structure.

Class Label	Description	Number of Images	File Format	Resolution (approx)
Carbonated Samples	Concrete exhibiting colorless phenolphthalein reaction (pH < 9)	431	.jpg	12 MP (cropped)
No Carbonation Presence	Concrete exhibiting pink phenolphthalein reaction (pH > 9)	472	.jpg	12 MP (cropped)
Metadata	Master inventory file with specimen details	1 (CSV/XLSX)	.csv/.xlsx	N/A
Total		903		

For consistency, a naming convention was adopted:

• Carbonated Samples follow the format: Carbonated_Sample (X).jpg
• Non-carbonated Samples follow the format: No_Carbonation_Presence (X).jpg

Images were captured using a mobile phone camera under varying angles, distances, lighting conditions, and orientations, ensuring diversity within each class and providing robustness for computer vision applications.

**Fig. 2:**
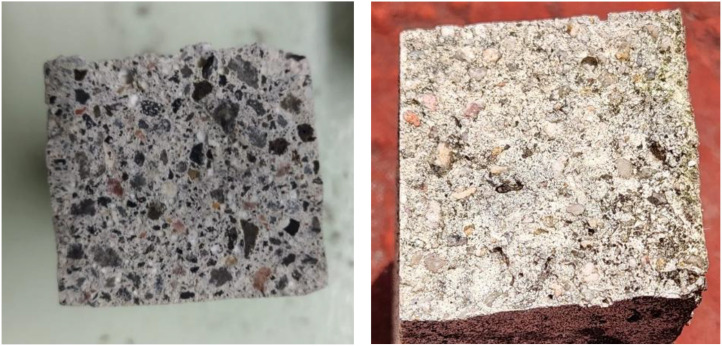
Representative images from the ConcreteCARB dataset.

**Fig. 3:**
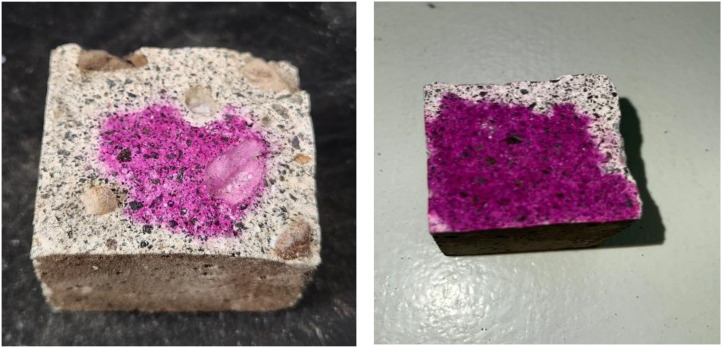
Concrete samples images contained in the dataset with No Carbonation Presence.

Representative examples of Carbonated Samples are shown in Fig. 2, while the samples with No Carbonation Presence are similar to those shown in Fig. 3.

The image dataset contains two folders representing the binary classes: “Carbonated Samples” and “No Carbonation Presence”. Classification is based on the phenolphthalein indicator test. Pink regions are labeled as “No Carbonation Presence” (pH > 9), and colorless regions as “Carbonated Samples” (pH < 9).

This dataset supports binary classification tasks. Quantitative measurements, such as carbonation depth or percentage of carbonated area, were not recorded. Labels indicate only the presence or absence of carbonation. Images are identified by sequential filenames.

A metadata file (Metadata.csv) accompanies the image dataset. It links each image filename to its Specimen ID, Mixture Type (1, 2, or 3), Water-Cement Ratio, Additives (industrial silica waste or Opuntia ficus-indica), test age (180 days), and exposure condition. Technical details, such as camera model (Samsung Galaxy S22) and approximate imaging distance, are also included.

## 4 Experimental Design, Materials and Methods

### 4.1 Experimental design

The quantitative and experimental design for the ConcreteCARB dataset was conducted under controlled laboratory conditions. The main goal was to create a high-quality image dataset of concrete carbonation damage to train and validate neural networks and transformers for automated carbonation detection. The experimental design involves the following elements:

• Objective: Generating and classifying Concrete Carbonated Samples and No Carbonation Presence to develop advanced neural network models, algorithms, and Graphic User interfaces (GUIs) for classification, detection, and segmentation tasks.
• Approach: A quantitative approach using image analysis for collecting and classifying carbonation damage on concrete samples.
• Design: An experimental design was conceived, involving controlled testing for developing the phenolphthalein test and then make a visual and manual classification of concrete Carbonated Samples.

The concrete samples were fabricated in the Laboratory “Ing. Luis Silva Ruelas” located at the Civil Engineering Faculty, at the “Universidad Michoacana de San Nicolás de Hidalgo”, Morelia, Michoacán, México.

### 4.2 Materials

**Fig. 4:**
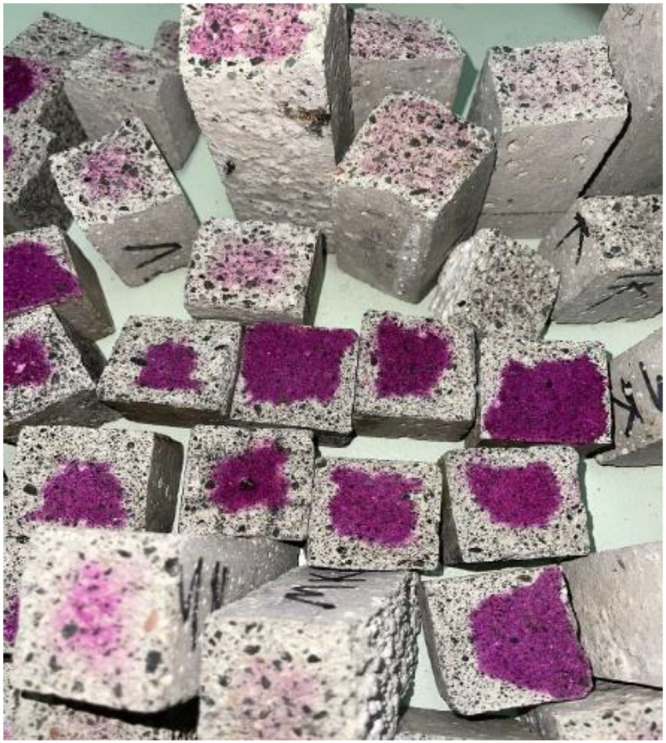
Concrete Samples beam elements tested to different depth.

ConcreteCARB dataset is composed of concrete samples images. The concrete elements analyzed are beams fabricated with the following dimensions: 4 cm x 4 cm x 16 cm (width, height, length). The concrete elements contain different concrete mix proportions as is described in Table 2. Examples of concrete beam elements with no presence of carbonation to different depths are show in Fig. 4.

**Table 2:** Mixtures composition and features of the ConcreteCARB dataset.

Mixture	Water-cement ratio	Additives	Test age (days)
Mixture-1	0.65	Industrial silica waste	180
Mixture-2	0.65	Opuntia ficus-indica nopal cactus (Proportion 1:2)	180
Mixture-3	0.60	Opuntia ficus-indica nopal cactus (Proportion 1:3)	180

### 4.3 Sample preparation of concrete specimens

Concrete prism samples were selected and cured under controlled laboratory conditions. Three different concrete mix designs were employed, varying the water–cement ratio (0.60 or 0.65) and incorporating specific additives (industrial silica waste, or a natural admixture derived from Opuntia ficus-indica nopal cactus in two mix proportions). All mixes used ordinary Portland cement and standard sand and gravel aggregates. After 24 h of initial set, specimens were demolded and placed in moist curing conditions for 28 days to ensure adequate hydration. Upon completion of curing, the concrete prisms were stored at ambient laboratory conditions until reaching the designated test age of 180 days. At this age, each sample’s compressive strength and maturity were sufficient for carbonation testing (as per durability guidelines in Mexican standards) [4].

### 4.4 Atmospheric carbonation process

At 28 days of age, the concrete specimens were subjected to an atmospheric carbonation process to monitor carbonation of their surfaces. The prisms were exposed to natural environmental conditions with continuous exposure to CO₂ in the Morelia Michoacán area. The atmospheric conditions were continuously monitored, given results as ranges of approximately 0.04 % CO₂ concentration, a temperature of 14–29 °C, and 40–70 % relative humidity. These environmental parameters are known to significantly speed up the natural carbonation process by providing a high CO₂ diffusion rate while avoiding excessive humidity that could hinder CO₂ ingress [5,6]. The samples remained exposed to the environmental conditions for several weeks (up to the 180-day total age), ensuring that a measurable carbonation front was developed from the exposed surfaces inward. This natural carbonation procedure aims to monitor long-term atmospheric carbonation. By the end of the exposure period, the concrete prisms had experienced varying extents of carbonation penetration depending on their mix composition (the different water–cement ratios and additives influence carbonation resistance).

### 4.5 Surface cutting and phenolphthalein test

**Fig. 5:**
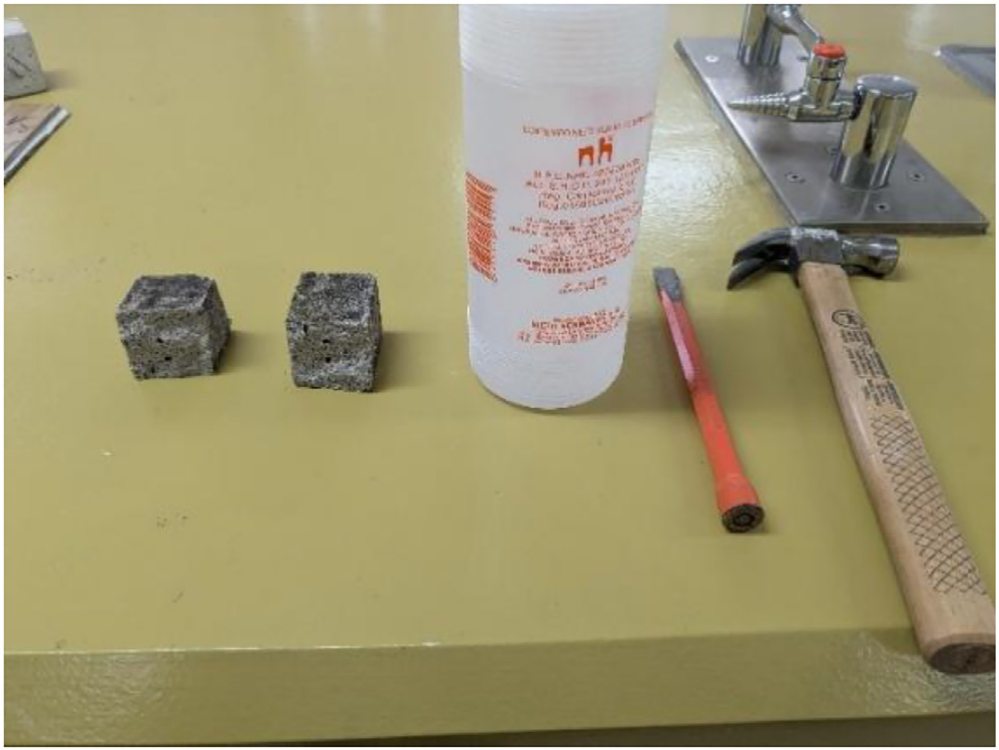
Chisel, hammer, and a 1 % phenolphthalein solution used for deploying the carbonation test.

After the carbonation exposure, each concrete specimen was cut to expose a fresh interior cross-section for analysis. To avoid heating or contaminating the surface (which could occur with saw cutting), the authors employed a manual chisel-and-hammer method. A chisel was placed at the mid-span of the prism and struck with a hammer, carefully propagating a crack and fracturing the sample into two pieces. This procedure produced a clean, freshly broken surface that had not been previously exposed to air. Immediately after splitting the specimen, the phenolphthalein indicator test was performed on the interior face. A 1 % phenolphthalein solution (phenolphthalein dye dissolved in 70 % ethyl alcohol and 30 % water) was sprayed evenly across the fresh concrete surface. The color change was observed over the next 60 seconds. All the used tools for deploying the phenolphthalein test are shown in Fig. 5.

Areas of the concrete that remained highly alkaline (i.e., not carbonated) turned a vivid pink/magenta color upon contact with the phenolphthalein solution. In contrast, areas that had experienced carbonation remained colorless. In practice, a purple-pink stain indicates the presence of intact calcium hydroxide (pH > ∼9–10) in the concrete, while a lack of color indicates that the pore water pH has dropped due to carbonation (pH < ∼9) [7]. This simple visual test thus reveals the depth of CO₂ penetration: a sharp boundary between pink and colorless regions marks the carbonation front. The phenolphthalein testing was carried out in accordance with relevant standards for carbonation depth measurement and is widely used in durability evaluations [8].

**Fig. 6:**
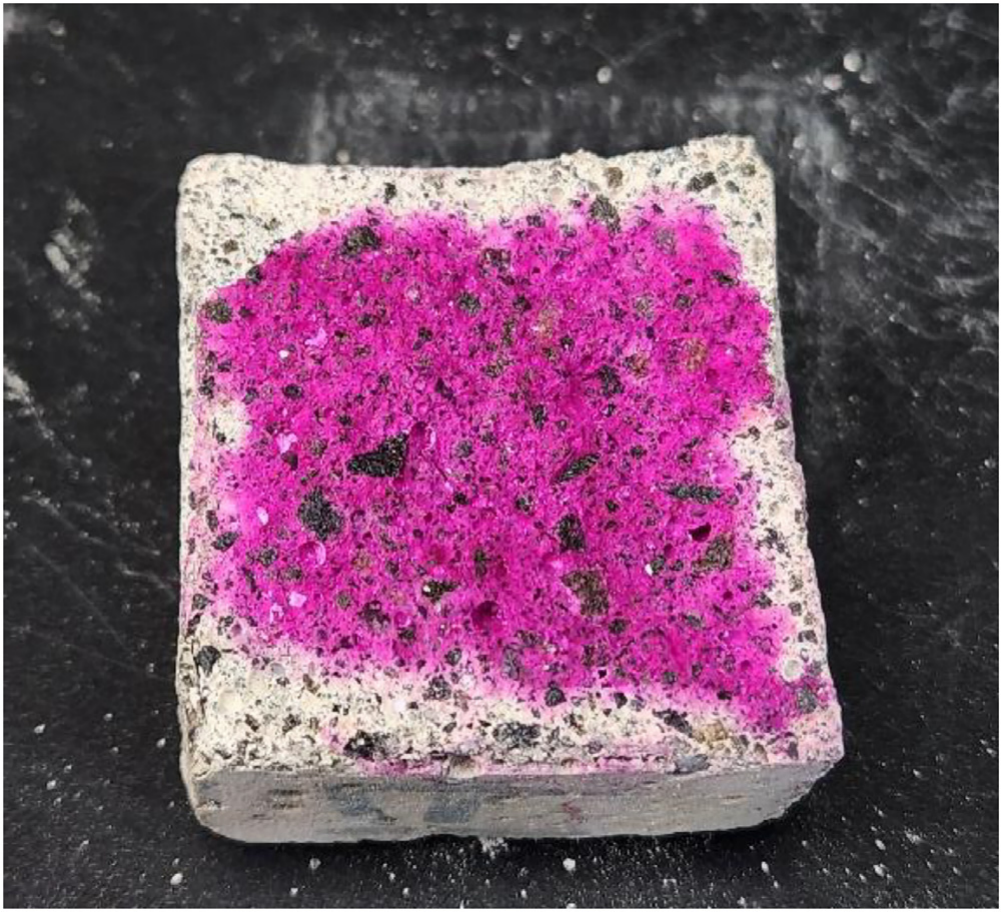
Sample of a carbonated specimen after the phenolphthalein test.

Fig. 6 shows an example of a Carbonated Sample after splitting and spraying with phenolphthalein, where the outer portion of the cross-section has no pink coloration (carbonated zone) and the inner core remains pink (uncarbonated concrete). All samples were tested in this manner to confirm the presence or absence of carbonation before imaging.

### 4.6 Image acquisition and processing

High-resolution digital photographs were taken of each tested concrete surface to create the ConcreteCARB image dataset. All images were captured using a Samsung Galaxy S22 (Model SM-S901U1) smartphone camera at an approximate resolution of 12 MP. The photography was conducted under controlled laboratory lighting using a 5000 K LED source to ensure consistent color reproduction of the phenolphthalein indicator. The camera was held approximately perpendicular to the specimen surface, with a maximum angular deviation of ≤ 7°, at a working distance of 30–50 cm. While original Exif metadata is retained for the majority of files, selected images were digitally cropped to exclude background elements (e.g., laboratory tools, operators) and focus exclusively on the concrete texture. Key camera settings included an f/1.8 aperture (a wide aperture for adequate light capture and a shallow depth of field), ISO 20 sensitivity (a low ISO to minimize noise), and an approximate focal length of 5 mm (a fixed wide-angle lens). Multiple angles and slight variations in distance were also employed across the dataset to introduce diversity in perspective, scale, and illumination. This approach ensures that the dataset includes a realistic range of imaging conditions, making trained AI models more robust to field conditions. All images were saved in JPG format with minimal post-processing (aside from standardizing file naming, as described earlier).

### 4.7 Data labelling and classification

Each captured image was manually reviewed and labelled by the authors, who have expertise in concrete materials and durability testing. The ground-truth label for each image was assigned based on the phenolphthalein test result visible in the photograph: if the concrete cross-section showed any colorless (unstained) region indicating carbonation, the sample was labelled as a “Carbonated Sample”. Contrarily, if the broken surface displayed a uniform pink coloration, it was labelled as “No Carbonation Presence”. This binary classification protocol was strictly followed for all 903 images. The use of the phenolphthalein chemical indicator ensures that the labelling correlates directly with the concrete’s chemical condition (alkaline vs. carbonated) rather than relying solely on subjective visual clues. Two of the authors independently verified the phenolphthalein color outcome for each sample to minimize the risk of mislabelling. The authors included only images with a clear, unambiguous test result in the final dataset; any specimens with indeterminate staining or testing anomalies were excluded to maintain high data quality. The resulting labelled image set was then organized into the two folder categories (“Carbonated_Samples” and “No_Carbonation_Presence”) as described, providing a structured dataset for subsequent computer vision model development.

## Limitations

The ConcreteCARB dataset was collected under controlled laboratory conditions, which may not fully represent carbonation patterns occurring in structures exposed to weathering. Images were captured with a mobile phone camera, potentially leading to variations in lighting, distance, and angle. The dataset includes only surface-level carbonation and a binary classification (Carbonated Samples vs. No Carbonation Presence), excluding intermediate or partial states. Additionally, despite careful manual verification, some subjective bias in labelling may persist. Users should consider these factors when applying the dataset for training, validation, or benchmarking of AI models in carbonation detection.

## Ethics Statement

The authors of this dataset meet the ethical requirements for publication in Data in Brief and do not involve human subjects, animal experiments, or any data collected from social media platforms.

## CRediT Author Statement

**José A. Guzman-Torres:** Conceptualization, Methodology, Writing-Reviewing and Editing, Data curation, Visualization, Investigation, Supervision. **Sandra del C. Arguello Hernández:** Data curation, Investigation, Methodology. **Francisco J. Domínguez Mota:** Visualization, Reviewing, Supervision. **Gerardo Tinoco-Guerrero:** Methodology, Writing-Reviewing Editing. **Elia M. Alonso Guzmán**: Investigation, Reviewing and Editing.

## Data Availability

Mendeley DataConcreteCARB (Original data)
